# Is Lymphedema a Systemic Disease? A Paired Molecular and Histological Analysis of the Affected and Unaffected Tissue in Lymphedema Patients

**DOI:** 10.3390/biom12111667

**Published:** 2022-11-11

**Authors:** Stefan Wolf, Julia von Atzigen, Bettina Kaiser, Lisanne Grünherz, Bong-Sung Kim, Pietro Giovanoli, Nicole Lindenblatt, Epameinondas Gousopoulos

**Affiliations:** Department of Plastic Surgery and Hand Surgery, University Hospital Zurich, 8091 Zurich, Switzerland

**Keywords:** secondary lymphedema, lymphatic system, inflammation, CD4+ cells, fibrosis, systemic changes

## Abstract

Secondary lymphedema is a chronic, debilitating disease and one of the most common side effects of oncologic surgery, substantially decreasing quality of life. Despite the progress conducted in lymphedema research, the underlying pathomechanisms remain elusive. Lymphedema is considered to be a disease affecting an isolated extremity, yet imaging studies suggest systemic changes of the lymphatic system in the affected patients. To evaluate potential systemic manifestations in lymphedema, we collected matched fat and skin tissue from the edematous and non-edematous side of the same 10 lymphedema patients as well as anatomically matched probes from control patients to evaluate whether known lymphedema manifestations are present systemically and in comparison to health controls. The lymphedematous tissue displayed various known hallmarks of lymphedema compared to the healthy controls, such as increased epidermis thickness, collagen deposition in the periadipocyte space and the distinct infiltration of CD4+ cells. Furthermore, morphological changes in the lymphatic vasculature between the affected and unaffected limb in the same lymphedema patient were visible. Surprisingly, an increased collagen deposition as well as CD4 expression were also detectable in the non-lymphedematous tissue of lymphedema patients, suggesting that lymphedema may trigger systemic changes beyond the affected extremity.

## 1. Introduction

Secondary lymphedema is a substantial side effect of oncologic treatment following iatrogenic damage of the lymphatic system or lymph node dissection in breast cancer [1] or other solid tumors, such as melanoma [2], sarcoma [3], gynecological [4] and urologic malignancies [5]. It is estimated that 20–40% of the patients undergoing oncologic surgery, involving lymphadenectomy for solid tumors, develop a measurable degree of lymphedema [6].

Lymphedema is clinically characterized by the localized progressive swelling of the affected extremity. Following lymphatic vascular injury, protein-rich lymph stagnates locally in the interstitial tissue, activating inflammatory pathways and causing fibro-adipose tissue accumulation. This explains why lymphedema usually appears months to years following initial lymphatic damage and becomes irreversible when fibrosis is established [7]. Recent research from various independent laboratories has revealed that chronic inflammation is a hallmark of lymphedema and a key driver of the observed fibrosis that occurs during the progression of the disease. In fact, the inflammatory composition in the rodent lymphedema models is characterized by a predominant CD4+ cell infiltrate, accounting for over 70% of the immune cell load [8,9]. Similar changes have been identified in human specimens collected from patients with unilateral upper extremity breast cancer-related lymphedema, where the number of tissue-infiltrating CD4+ T cells positively correlated with the severity of the disease [10]. Importantly, immunomodulation in experimental lymphedema models has shown to reduce lymphedema development, indicating the causal role of inflammation in the onset and development of the disease [8,11,12].

Despite lymphedema being considered a localized disease affecting the extremities, where the draining lymph nodes have been removed, recent work has illustrated the complexity of the disease, with systemic abnormalities and cytokine changes in lymphedema patients. Current studies have shown that global abnormities in the lymphatic function are detected in breast cancer-related lymphedema patients. These patients also have a higher pumping pressure in their contralateral arms than the patients who do not develop lymphedema, indicating that lymphedema progression has a systemic and not just a regional effect [13]. Furthermore, global abnormalities in lymphatic vascular function were reported in lymphedema patients, which indicates factors that may predispose patients to develop lymphedema or systemic changes during the course of the disease [14,15].

In this study, we compared matched biopsies of edematous and non-edematous tissue from secondary lymphedema patients to anatomically matching control (healthy) patient biopsies in order to evaluate whether changes occurring in the lymphedematous tissue result into systemic changes manifesting in distant tissues. Our analysis of lymphedematous tissue confirmed the known lymphedema hallmarks. Surprisingly though, an increased collagen deposition and increased expression level of CD4 were also found in the non-edematous tissue from lymphedema patients, suggesting systemic changes in lymphedema patients. These findings imply that lymphedema is not a disease confined to the affected extremity but also triggers systemic effects in tissues distant to the affected limbs.

## 2. Materials and Methods

### 2.1. Study Population

The protocols of the current study were approved by the Ethical Committee of the Canton of Zurich KEK-ZH: StV 7-2009), the Swiss ethics (BASEC-Nr.: 2019-00389), and the study was conducted according to the principles of the Declaration of Helsinki. All patients were informed prior to the surgical procedures in oral and written form, and they then provided their written informed consent. Tissue was collected from lymphedema patients undergoing elective surgery with autologous lymph node transfer. In that regard, healthy tissue (non-lymphedematous) was obtained from an unaffected location, where the lymph node was harvested. Lymphedematous tissue was harvested from the location where the lymph node transplantation occurred. Thus, matched biopsies from edematous and non-edematous locations from the same patients were obtained. For the transfer to the leg, lymph nodes were harvested from the axilla and lymph nodes for the arm from the groin. Healthy control patients served the patients who underwent elective surgeries, had a matching BMI and could provide anatomically matching fat and skin samples. General patient characteristics are provided in Table 1 and detailed patient information in Supplementary Table S1.

### 2.2. Tissue Collection and Histology

During the operating procedure, fat tissue and skin specimens for histology were collected and fixed in paraformaldehyde/phosphate-buffered saline (PBS) at 4 °C. Subsequently, the samples were embedded in paraffin. For the histological analysis and assessment of adipose and skin tissue architecture, the specimens were cut into 5 μm-thick paraffin sections and stained at the Center for Surgical Research of the University Hospital Zurich for hematoxylin/eosin (H/E) and Sirius Red (SR), in accordance with previously published protocols [16].

### 2.3. Immunohistochemistry

For the immunohistochemical stainings, paraffin-embedded sections were deparaffinized and rehydrated. Stainings were performed using the Autostainer Link48 (DAKO). For the antigen retrieval of stainings with CD45 (monoclonal mouse anti human, IR751, Dako), CD68 (monoclonal mouse anti human, IR613, Dako) and CD4 (monoclonal mouse anti human, IR649, Dako), Target Retrieval Solution high ph9 (Dako K8004) was used; for Podopanin (monoclonal mouse anti human, IR072, Dako), Target Retrieval Solution high pH6.0 (DakoK8005) was used. Endogenous peroxidase activity was blocked using 3% Hydrogen Peroxide (Merck, Darmstadt, Germany). After blocking with goat serum for 30 min at RT, the sections were incubated with primary antibodies in RTU dilution. After the washing steps with PBS, the bound antibody was visualized using the DAB substrate (Dako K3468), according to the manufacturer’s instructions.

Histology images were obtained using a Zeiss Axio Scan Z1 equipped with a Hitachi HV-F202FCL, and the whole tissue section was scanned using a Plan Apochromat 20×/0.8 numerical aperture objective.

For the histological evaluation, five random ROIs were placed. The exported morphometric analysis of adipocyte characteristics and fibrosis quantification with Sirius Red was performed using ImageJ software (National Institutes of Health, Bethesda, MD, USA). Fibrosis quantification was performed at a constant color threshold and assessed as a percentage of red staining/tissue surface ratio.

### 2.4. RNA Extraction and Quantitative Polymerase Chain Reactions

Fat tissue was collected during the operating procedure and immediately flash-frozen in liquid nitrogen. RNA was isolated from a 100 mg piece of fat tissue using the RNeasy Lipid Tissue Mini Kit (Qiagen, Hilden, Germany). Complementary DNA was transcribed from a 500 ng RNA template, using the High-Capacity cDNA Reverse Transcription Kit (ThermoFisher Scientific, Waltham, MA, USA). The polymerase chain reactions were performed using Fast SYBR™ Green Master Mix (Applied Biosystems, Waltham, MA, USA) and QuantStudio 5 Real-Time PCR Systems. B2M was used as a housekeeping gene, and the fold changes of the gene expression were calculated using the ΔΔCT method.

### 2.5. Statistics

The statistical analysis was performed using GraphPad Prism V 8.0 (GraphPad Software, San Diego, CA, USA). All data represent the mean ± SD, as depicted in whisker plots exhibiting the 5–95 percentiles. In order to compare the edematous and non-edematous tissue from the same patient, a paired Student’s t-test was used; for the comparison of two samples from the lymphedema to the healthy control tissue, a one-way ANOVA followed by a Fischer’s LSD test was performed. For the analysis of the adipocyte size distribution, a Fisher’s exact test was performed. *p* < 0.05 was accepted as statistically significant.

## 3. Results

### 3.1. Increased Epidermis Thickness in Lymphedema Patients

**Figure 1:**
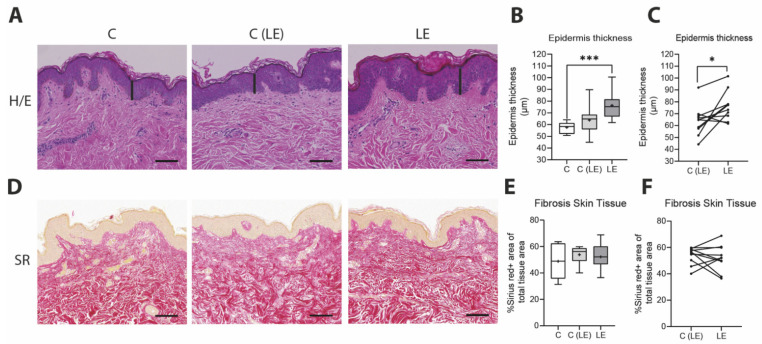
Increased epidermis thickness without increased fibrosis in skin in lymphedema. (**A**) Hematoxylin/eosin (H/E) stains from paraffin-embedded skin. The black lines indicate the epidermal thickness. (**B**) Quantification of epidermal thickness from hematoxylin/eosin stains, depicting significantly increased epidermal thickness in the lymphedema-affected extremity. (**C**) A paired comparison of the epidermal thickness of non-edematous and edematous skin tissue in lymphedema indicates significantly higher epidermal thickness in the lymphedema-affected area in comparison to distant locations (**D**) Sirius Red (SR) stains from paraffin-embedded skin were used to evaluate the collagen deposition in red. (**E**) Collagen content was quantified from Sirius Red sections (**F**) A paired comparison of collagen content in non-edematous and edematous skin tissue in lymphedema suggests no significant changes in dermal collagen deposition. Scale bar: 100 µm. Asterisks indicate statistical significance; * *p* < 0.05 and *** *p* < 0.001 (for the comparison with the healthy control, ANOVA followed by a Fischer’s LSD test was used. For the comparison of the edematous and the non-edematous tissue from the same patient, a paired Student’s *t*-test was used). C: Control, C(LE): control from non-lymphedematous tissue from lymphedema patients, LE: lymphedematous tissue.

In our study, we compared biopsies of lymphedematous and non-lymphedematous tissue from patients with secondary lymphedema versus anatomically matching control patients. Changes in skin thickness have been shown to correlate with lymphedema severity [17]. In our study, an increased epidermal thickness was observed in the edematous tissue of lymphedema patients (healthy control [C]: 57.55 ± 4.80 µm versus non-lymphedematous tissue [C(LE)]: 63.78 ± 12.70 µm versus lymphedematous tissue (LE): 76.39 ± 12.4 µm). The significant increase was visible in the unpaired analysis compared to the control group (*p* = 0.0008) as well as the paired comparison between the edematous and non-edematous tissue of lymphedema patients (*p* = 0.02). Cutaneous fibrotic tissue deposition was assessed by the quantification of Sirius Red staining, which showed no significant alteration (Figure 1).

### 3.2. Increased Fibroadipose Tissue in Lymphedema Patients

It is well established that an increased deposition and remodeling of adipose tissue characterized by hypertrophy occurs in the early years of lymphedema onset [18]. These clinical observations, as reported by Zhang et al. [19], have been further confirmed in lymphedema experimental models—defined as increased lipid content and the expression of adipogenesis-related genes [20,21].

**Figure 2:**
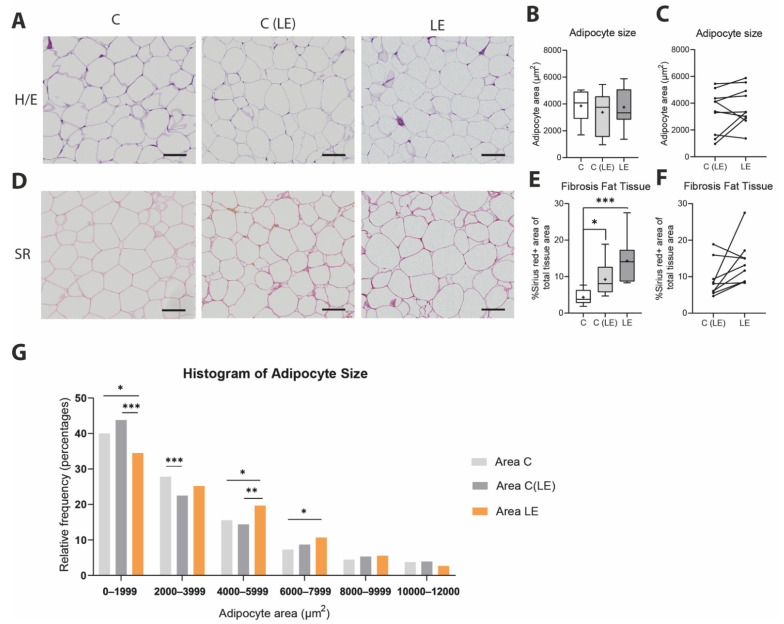
Increased fibrosis in lymphedema. (**A**,**D**) Hematoxylin/eosin (H/E) and Sirius Red stains (SR) of paraffin-embedded fat sections were used to evaluate the collagen deposition stained in red. (**B**) Quantification of the adipocyte size among the three groups using the H/E sections revealed no changes in the adipocyte size. (**C**) A paired comparison of the adipocyte size in non-edematous and edematous fat tissue in lymphedema reveals comparable adipocyte size in both locations. (**E**) Quantification of collagen deposition was performed using SR-stained sections, indicating increased collagen deposition in both the non-edematous and edematous tissue in lymphedema. (**F**) A paired comparison of collagen content in non-edematous and edematous fat tissue in lymphedema reveals a trend towards increased fibrosis, without reaching statistical significance. (**G**) Histogram presentation of the adipocyte size ranges of the lymphedematous, non-lymphedematous and healthy control adipose tissue. The analysis reveals a comparable size distribution across the three groups but significantly higher numbers of large adipocytes in lymphedematous tissue. Scale bar: 100 µm. Asterisks indicate statistical significance; * *p* < 0.05, ** *p* < 0.01 and *** *p* < 0.001. (For the comparison to the healthy control, an ANOVA test followed by a Fischer’s LSD test was used. For the comparison of the edematous and the non-edematous tissue from the same patient, a paired Student’s *t*-test was used. For the adipocyte size distribution, a Fisher’s exact test was performed). C: Control, C(LE): control from non-lymphedematous tissue from lymphedema patients, LE: lymphedematous tissue.

In order to verify these findings in our patient cohort, we evaluated the adipose tissue morphology and degree of fibrotic tissue deposition in a histological analysis using H/E and SR stains, respectively. The evaluation of the adipocyte size across the three different groups revealed no significant differences in the average adipocyte size (Figure 2A–C). The assessment of the size distribution for the adipocytes revealed higher numbers of larger adipocytes in the lymphedematous extremities versus the other two conditions (Figure 2G). Finally, the quantification of SR stains showed significantly increased collagen deposition among the adipocytes from the edematous as well as from the non-edematous tissue versus the healthy control biopsies from anatomically and BMI-matched patients (Figure 2C–E; healthy control [C]: 4.33 ± 2.05% versus non-lymphedematous tissue [C(LE)]: 9.21 ± 4.94 versus lymphedematous tissue (LE): 14.28 ± 5.79% C vs. C(LE) *p* = 0.020; C vs. LE *p* = 0.003).

### 3.3. Morphological Changes in the Lymphatic Vasculature in Lymphedema

**Figure 3:**
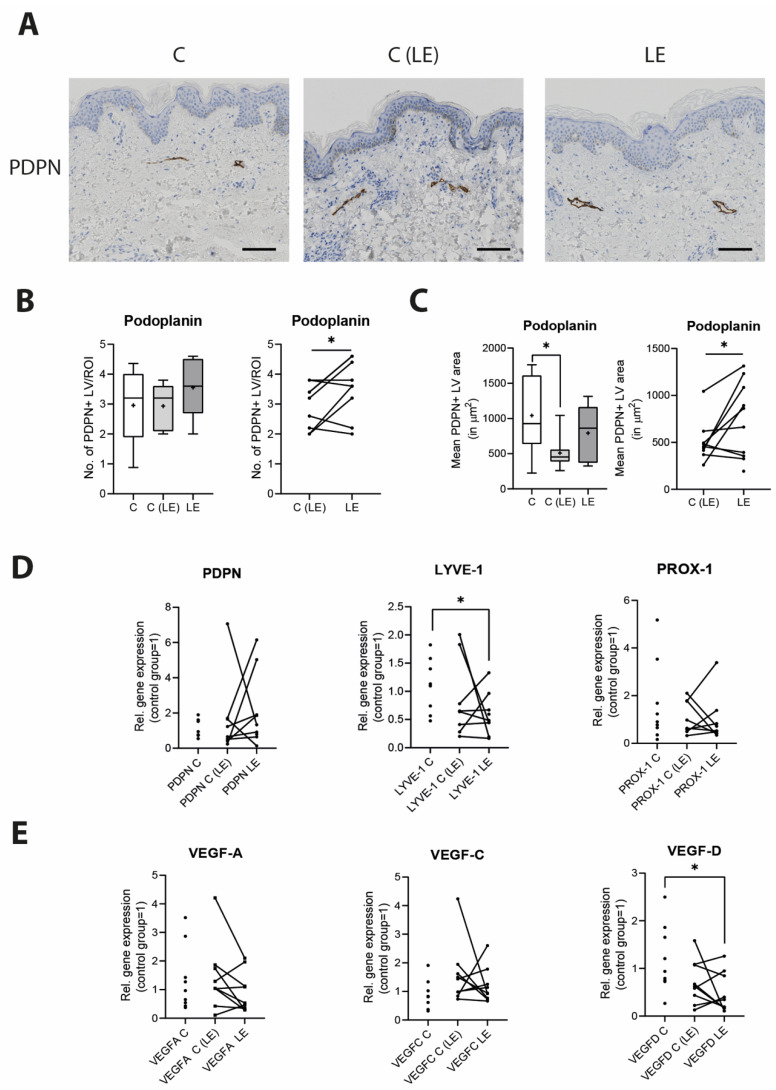
Morphological and gene-expression changes of lymphatic vessels in lymphedema. (**A**–**C**) Histological evaluation of the lymphatic vessels on skin sections using podoplanin/PDPN revealed distinct changes in the number, size of lymphatic vessels between tissue from control tissue, non-lymphedematous and lymphedematous tissue from lymphedema patients, with a significant increase in the number and size of lymphatic vessels in lymphedematous tissue. (**D**,**E**) The evaluation of the PDPN, LYVE-1, PROX-1, VEGF-A, VEGF-C and VEGF-D expression in fat tissue decreased the LYVE-1 and VEGF-D expression in the lymphedematous tissue. Scale bar: 100 µm. Asterisks indicate statistical significance; * *p* < 0.05 (for the comparison to the healthy control, an ANOVA followed by a Fischer’s LSD test was used. For the comparison of the edematous and the non-edematous tissue from the same patient, a paired Student’s *t*-test was used). C: Control, C(LE): control from non-lymphedematous tissue from lymphedema patients, LE: lymphedematous tissue.

As significant changes occur in the lymphatic vascular morphology during the course of the disease, we next performed a detailed histological analysis of lymphatic vasculature by using the lymphatic marker podoplanin/PDPN to characterize the size and number of lymphatic vessels in paraffin tissue sections. Although no changes in regard to the number of lymphatic vessels were identified among the three groups (healthy control [C]: 2.96 ± 1.15 vessels/field versus non-lymphedematous tissue [C(LE)] 2.93 ± 0.74 vessels/field versus lymphedematous tissue [LE] 3.48 ± 0.93 vessels/field), the paired analysis between the lymphedematous and non-lymphedematous groups indicated a slight but statistically significant (*p* = 0.047) increase in the number of lymphatic vessels in the lymphedematous extremity (Figure 3A,B). The average lymphatic coverage per ROI is displayed in Supplementary Figure S1.

Furthermore, the comparison of the mean lymphatic vessel coverage across the three groups revealed a significant reduction in coverage in the non-lymphedematous extremity, (*p* = 0.0141) and a trend towards an increased coverage in lymphedematous extremities in comparison to the non-lymphedematous extremities, without reaching significance (healthy control [C]: 1042 ± 541 μm^2^ versus non-lymphedematous tissue [C(LE)] 508 ± 223 μm^2^ versus lymphedema [LE] 791 ± 379 μm^2^). However, in the paired analysis, a significantly increased lymphatic vessel coverage was detected in the lymphedematous versus non-lymphedematous probes (*p* = 0.049) (Figure 3A–C).

We further analyzed the potential alterations of the lymphatic vasculature by evaluating the expression profile of the most common lymphatic vessel-related genes in the adipose tissue using qPCR. No significant changes in the expression patterns were observed for most of the common lymphatic markers—namely, PDPN, PROX-1, VEGF-A and VEGF-C—but we detected a 0.489-fold expression decrease in LYVE-1 (*p* = 0.024) and a 0.379-fold expression decrease in VEGF-D (*p* = 0.021) in the edematous lymphedema tissue compared to the healthy control (Figure 3D,E).

### 3.4. Systemic Increase in CD4+ T Cells in Lymphedema

**Figure 4:**
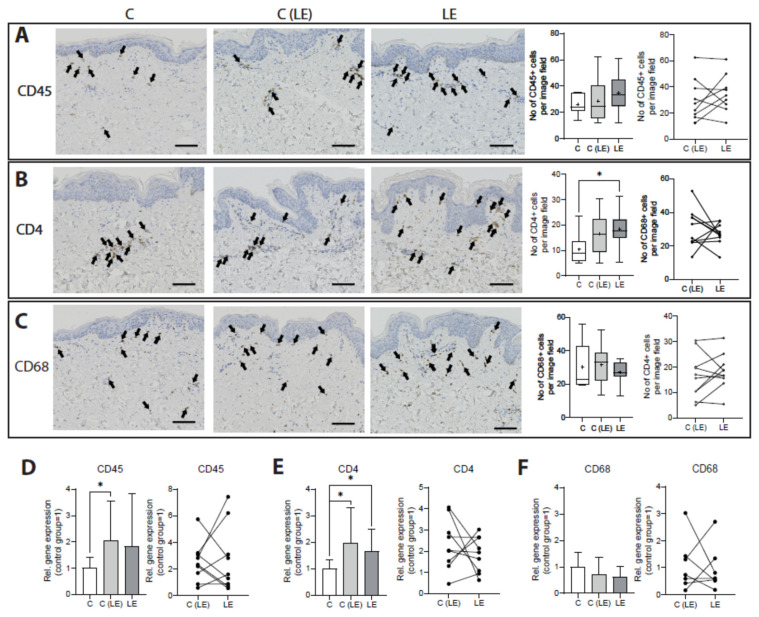
An increased CD4+ cell infiltrate characterizes lymphedema. (**A**–**C**) The immune cell infiltration of CD45+ (leucocytes), CD4+ (CD4+ T helper cells) and CD68+ (macrophages) cells was evaluated on paraffin-embedded tissue sections. The arrows indicate the CD45-, CD4- and CD68-positive cells, respectively. The quantification of the CD45+, CD4+ and CD68+ cells reveals an increased infiltration of CD4+ cells in the lymphedematous tissue. (**D**–**F**) The evaluation of the CD45, CD4, CD68 expression in fat tissue. Scale bar: 100 µm. Asterisks indicate statistical significance; * *p* < 0.05. For the comparison with the healthy control, an ANOVA followed by a Fischer’s LSD test was used. For the comparison of the lymphedematous and the non-lymphedematous tissue from the same patient, a paired Student’s *t*-test was used. C: Control, C(LE): control from non-lymphedematous tissue from lymphedema patients, LE: lymphedematous tissue.

As the role of the immune cell compartment has been undoubtedly proven to decisively influence lymphedema in experimental models, we next sought to characterize the immune cell composition in paraffin-embedded skin sections. CD45 was used to evaluate the total leucocyte infiltration, whereas CD4 is a marker for CD4+ T helper cells and CD68 for macrophages. No changes in the number of CD45+ (healthy control [C]: 26.1 ± 7.5 cells/field versus non-lymphedematous tissue [C(LE)] 28.7 ± 16.2 cells/field versus lymphedematous tissue [LE] 34.8 ± 14.5 cells/field) and CD68+ (healthy control [C]: 29.4 ± 13.3 cells/field versus non-lymphedematous tissue [C(LE)] 32.5 ± 11.7 cells/field versus lymphedematous tissue [LE] 27.6 ± 6.5 cells/field) cell infiltration were detected. A significant increase in CD4+ cells in the edematous lymphedema tissue versus the control was noted (*p* = 0.028) (healthy control [C]: 10.5 ± 5.8 cells/field versus non-lymphedematous tissue [C(LE)] 16.4 ± 8.8 cells/field (C vs. (C(LE) *p* = 0.089) and lymphedematous tissue [LE] 18.3 ± 6.9 cells/field), while the number of CD4+ cells was found to be comparable between the non-lymphedematous and lymphedematous tissue of the lymphedema patients (Figure 4A–C). Furthermore, we further evaluated the CD45, CD4 and CD68 gene expression in the subcutaneous fat tissue. A significant increase in the CD45 gene expression was detected in the non-edematous tissue (2.05-fold; *p* = 0.048), and a strong trend towards an increased CD45 expression in lymphedematous tissue (1.85-fold; *p* = 0.088) was compared with the healthy control. Very interestingly, CD4 was significantly more expressed in both specimens from lymphedema patients (C(LE): 1.98-fold, *p* = 0.011 and LE: 1.66-fold; *p* = 0.043). In the paired analysis of lymphedematous versus non-lymphedematous tissue, the expression of both CD45 and CD4 was found to be comparable. The expression of CD68 among the three groups did not reveal any changes (Figure 4D–F).

## 4. Discussion

Lymphedema is an immense burden for the individual, as well as for healthcare system. Despite the recent progress in research, the underlying pathomechanisms still remain elusive, which subsequently results in the delayed diagnosis, inefficient monitoring and eventual absence of a pharmacological treatment for lymphedema.

Lymphedema develops in a delayed fashion, months or years after the initial lymphatic injury, and affects only a fraction of patients undergoing lymph node removal. Furthermore, lymphedema’s phenotype is variable, ranging from limited swelling which is restricted to parts of the limb, to uniform swelling of the entire limb. Thus, the obstruction of the lymph drainage route is not a sufficient explanation for lymphedema onset, and research in recent years suggests that lymphedema may not be restricted to the affected extremity [13]. Stanton and his colleagues were the first researchers who showed that after axillary surgery and before the onset of edema, women who later develop breast cancer-related lymphedema have higher lymph flows than women who do not develop lymphedema [22]. Interestingly, the lymph flow was also found to be increased in the contralateral arm [13]. Similarly, lymphatic abnormalities have also been detected in another study in the contralateral arm of breast cancer-related lymphedema patients by near-infrared fluorescent imaging [23]; in addition, systemic abnormalities in lymphatic function with higher pumping pressures were detected in women destined to develop breast cancer-related lymphedema. Furthermore, Jensen et al. described a two-fold-higher capillary filtration coefficient value in non-edematous forearms than those of the control subjects [24]—a finding that was confirmed in experimental lymphedema models [25]. These findings indicate systemic factors that either predispose lymphedema or occur upon lymphatic vascular damage beyond the affected extremity [14,15].

Hence, in this study, we aimed to further evaluate these potential systemic lymphedema manifestations by comparing skin and fat tissue from the lymphedematous and non-lymphedematous sides of the same lymphedema patients to anatomically matched probes from gender- and BMI-matched control patients. A number of well-known histological hallmarks were identified in the lymphedematous tissue. First of all, increased epidermal thickness was identified in the edematous tissue [17,19,26], which is used as a primary outcome measure for the efficacy of lymphedema treatments, such as the anti-inflammatory drug ketoprofen or anti-Th2 immunotherapy [10,27]. Fibrosis, a well-known hallmark and key driver of lymphedema, was significantly increased in the lymphedematous adipose tissue, but to our surprise, increased collagen deposition was noted in the non-lymphedematous tissue as well. This suggests that factors contributing to fibrotic tissue deposition are present outside of the affected tissue milieu as well.

Lymphatic vessel hyperplasia and the modification of the lymphatic vascular architecture are integral parts of lymphedema progression. Both an increased number and increased size of lymphatic vessels were detected in the lymphedematous tissue, confirming previous findings. Interestingly though, the number of lymphatic vessels of the lymphedematous tissue was found to be comparable to the control tissue, while the size of the lymphatic vessels in the non-lymphedematous tissue was found to be decreased in comparison with the controls. The reason for such a finding might probably lay on the stage of lymphedema. In the early lymphedema stage, lymphatic vessels appear dilated due to the increased endolymphatic pressure. As lymphedema progresses, the constant increased pressure leads to a compensatory increase in smooth muscle cells and fibrosis, resulting in a functional decline and reduced lumen of the lymphatic vessels [28]. In our study, samples from stage I to stage III are included. Potential alterations could be addressed by analyzing all stages together, and a larger study population would be needed to evaluate the samples of each stage separately. Regarding the findings of the expression analysis, while a decreased LYVE-1 expression present in the lymphedematous tissue potentially occurs in conditions of inflammation, a decreased VEGF-D expression might act as a compensatory mechanism for the increased vascular permeability, as shown previously [25].

The accumulation of fibrotic tissue is tightly connected to the infiltration of CD4+ cells, which are a hallmark of lymphedema [10] and highly present in lymphedematous tissues in our study. Surprisingly though, the non-lymphedematous tissue of lymphedema patients displayed an increased CD4 expression as well. Furthermore, in the paired analysis of the affected and unaffected extremity of the same patient of many immune cell and lymphatic markers, no differences were detectable, indicating that systemic alterations are present in response to lymphedema development.

Our results indicate that the hallmarks of lymphedema are not limited to the edematous tissue in lymphedema patients. In recent years, more and more evidence has arisen which points in a similar direction, such as the alteration in plasma cytokine levels and the content of immune cells in the blood. In this regard, systemically elevated serum VEGF-C levels have been previously described in a lymphedema mouse model promoting blood vascular leakage [25]. Importantly, Jensen at al. discovered that systemically elevated VEGF-C levels are present in breast cancer-related lymphedema patients and are associated with an increased forearm capillary filtration capacity [29]. A recent study evaluated the systemic effect of lymphedema and detected higher lymphocyte numbers in the peripheral blood, with several deregulated miRNAs in the serum from lymphedema patients [30]. In further support of these findings, Lin et al. identified, in the serum of lymphedema, several altered proteins which are associated with lymphangiogenesis, inflammation, fibrosis and adipocytokine signaling [31].

The work in this study is complementary to observations from various independent groups over the last decade and indicates that the picture of lymphedema should be reviewed critically. This approach would improve the sensitivity of the diagnostic effort, which is currently based mostly on a clinical examination and a volume increase beyond 20% between the afflicted and unaffected arm, which results in a delayed diagnosis, particularly when not diagnosed by specialists. Changes in the lymphatic vasculature, immune cell composition or systemic factors that are attributed to their modification could be detectable earlier than the volume increase itself, which could improve the therapeutic intervention and monitor the progress of the disease [32,33].

The current study has been limited by the relatively small number of patients included due to the difficulty in receiving matched samples from the lymphedematous and non-lymphedematous locations of the same patient. Thus, samples from all lymphedema stages had to be included, which might present an additional limitation, which is in part counterbalanced by the paired analysis conducted. Despite this heterogeneity, which is especially visible in the probes from the unaffected limb, we could detect significant changes in comparison to the healthy control group. Furthermore, a set of parameters, such as the number of CD4+ cells in the skin of the unaffected limb or the CD45+ expression in the edematous tissue, showed a strong but not significant tendency, which further supports our hypothesis. Of course, such conclusions need to be validated in a larger study.

## 5. Conclusions

Together, our data revealed that lymphedema patients present increased fibrotic tissue deposition and an increased CD4 expression in distant tissue, which implies that lymphedema is not a disease confined to the affected extremity but also triggers systemic effects in tissues distant to the affected limbs.

## Figures and Tables

**Table 1:** Patient Characteristics.

Patient Characteristics	Lymphedema Patients	Control Patients
Number of cases	10	10
Gender		
Female	8	7
Male	2	3
Mean age (in years)	58.10 ± 10.86	43.18 ± 18.77
Mean BMI (in kg/m^2^)	28.02 ± 5.55	26.54 ± 5.63
Lymphedema stage		
Stage I	1	
Stage II	6	
Stage III	3	
Affected extremity		
Leg	8	
Arm	2	

## Data Availability

Not applicable.

## Supplementary Materials

The following supporting information can be downloaded at: https://www.mdpi.com/article/10.3390/biom12111667/s1, Figure S1: Histological evaluation of the lymphatic vessels on skin sections using podoplanin/PDPN revealed that the total lymphatic coverage in lymphedematous compared to the non-lymphedematous tissue from lymphedema patients is significantly increased; Table S1: Lymphedema patient characteristics; Table S2: Control patient characteristics; Table S3: Primer List.
