# Supplementary material for: Is Lymphedema a Systemic Disease? A Paired Molecular and Histological Analysis of the Affected and Unaffected Tissue in Lymphedema Patients
Source: Biomolecules. 2022 Nov 11;12(11):1667. doi: 10.3390/biom12111667 (PMC9687735; doi:10.3390/biom12111667)
Supplement: Supplementary file 1 [file biomolecules-12-01667-s001.zip › biomolecules-1977418-supplementary.pdf]

Supplementary

Suppl. Table S1: Lymphedema patient characteristics

Suppl. Table S2: Control patient characteristics

Suppl. Table S3: Primer List

Suppl. Figure S1:

Table S1: Lymphedema patient characteristics

Patient LE	Age	Gender	Affected Limb	Matched-biopsy location	Etiology	Clin. Stage	BMI (kg/m2)	FD (M/Y)
LE1	69	F	Left leg	Right trunk/ axilla	Uterus Ca	III-IV	29.8	2011
LE2	64	M	Left leg	Right trunk/ axilla	Melanoma	II	31.4	2014
LE3	66	F	Left leg	Right trunk/ axilla	Endometrial Ca	II	25.9	2007
LE4	57	F	Left leg	Right trunk/ axilla	Uterus Ca	III	24.3	2014
LE5	44	M	Left leg	Right trunk/ axilla	Melanoma	I	36	2017
LE6	63	F	Right leg	Left trunk/ axilla	Ovarial-Ca	III	23.6	2018
LE7	64	F	Left leg	Right trunk/ axilla	Infection upon sectio	II	22	2003
LE8	68	F	Left arm	Right groin	Mamma-Ca	II	36.9	2019
LE9	47	F	Left arm	Right groin	Mamma-Ca	II	29	2013
LE10	39	F	Right leg	Left trunk/ axilla	Trauma	II	21.3	2004

Table S2: Control patient characteristics

Patient C	Age	Gender	Location	Clin. Stage	BMI (kg/m2)	FD (M/Y)
C1	18	F	Thigh/ leg	NA	23	NA
C2	28	M	Abdomen/ groin	NA	24.9	NA
C3	53	F	Trunk	NA	22.4	NA
C4	48	F	Trunk	NA	25	NA
C5	51	F	Trunk	NA	26.8	NA
C6	57	M	Abdomen/ groin	NA	34.4	NA
C7	55	M	Abdomen/ groin	NA	32.9	NA
C8	27	F	Trunk	NA	35	NA
C9	45	F	Trunk	NA	22	NA
C10	16	F	Thigh/ leg	NA	19	NA

Table S3: Primer List

B2M forward	TGTGCTCGCGCTACTCTCTCT
B2M reverse	CGGATGGATGAAACCCAGACA
VEGF A forward	CTACCTCCACCATGCCAAGT
VEGF A reverse	GCAGTAGCTGCGCTGATAGA
VEGF C forward	CACCACCAAACATGCAGCTG
VEGF C reverse	TGAAAATCCTGGCTCACAAGC
VEGF D forward	ATGGACCAGTGAAGCGATCAT
VEGF C reverse	GTTCCTCCAAACTAGAAGCAGC
PDPN forward	AGGCGGCGTTGCCAT
PDPN reverse	GTCTTCGCTGGTTCCTGGAG
LYVE-1 forward	AGCTATGGCTGGGTTGGAGA
LYVE-1 reverse	CCCCATTTTTCCCACACTTG
PROX1 forward	ACAAAAATGGTGGCACGGA
PROX1 reverse	CCTGATGTACTTCGGAGCCTG
CD4 forward	CCTCCTGCTTTTCATTGGGCTAGG
CD4 reverse	TGAGGACACTGGCAGGTCTTCT
CD45 forward	CTTCAGTGGTCCCATTGTGGTG
CD45 reverse	CCACTTTGTTCTCGGCTTCCAG
CD68 forward	CGAGCATCATTCTTTCACCAGCT
CD68 reverse	ATGAGAGGCAGCAAGATGGACC

Suppl. Figure S1:

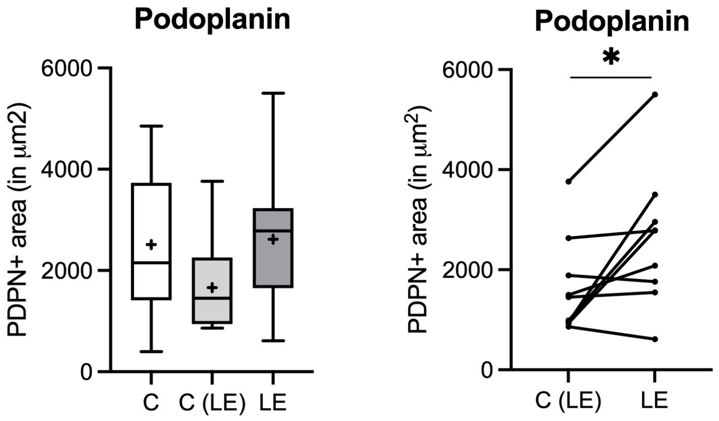

Figure S1: Histological evaluation of the lymphatic vessels on skin sections using podoplanin/PDPN revealed that the total lymphatic coverage in lymphedematous compared to the non-lymphedematous tissue from lymphedema patients is significantly increased. No alterations to the healthy control was detected. Asterisks indicate statistical significance; *$P < 0.05$ (For the comparison to the healthy control and ANOVA followed by a Fischer's LSD test was used. For the comparison of the edematous and the non-edematous tissue from the same patient, a paired Student's t-test was use). C: Control, C(LE): control from non-lymphedematous tissue from lymphedema patients, LE: lymphedematous tissue
